# Estimation of Humidity Variation and Electric Resistivity in Hardened Concrete by Means of a Stainless Steel Voltammetric Sensor

**DOI:** 10.3390/s22197279

**Published:** 2022-09-26

**Authors:** Ana Martínez Ibernón, Josep Lliso Ferrando, Isabel Gasch, Manuel Valcuende

**Affiliations:** 1 Interuniversity Research Institute for Molecular Recognition and Technological Development (IDM), Universitat Politècnica de València, 46022 Valencia, Spain; 2 Department of Architectural Construction, Universitat Politècnica de València, 46022 Valencia, Spain

**Keywords:** voltammetric sensors, monitoring structures, humidity estimation, electrical resistivity, corrosion

## Abstract

Reinforced concrete structures’ (RCSs) ageing and early deterioration are some of the main challenges faced by the building sector today, and steel bar corrosion is one of the main problems. In this phenomenon, water and concrete’s electric resistivity play a fundamental role. Therefore, developing sensor systems capable of estimating any variations in these parameters in real time and remotely would represent considerable progress in sustainably maintaining RCSs. Many types of sensors capable of estimating humidity variation and electrical resistivity in concrete currently exist, but the variability of these sensors’ sensitivity can be extreme depending on several factors; for example, temperature or presence of ions and their incorporation into smart monitoring systems, which is difficult. As an alternative to today’s sensors, this study centered on developing two estimation models by means of the response of a novel voltammetric stainless steel (SS) sensor. The estimation models were one of humidity variation and another of concrete’s electric resistivity. These models were calibrated, fitted and validated. In the validation, both these models explained a percentage of variance over 80%.

## 1. Introduction

Given today’s socio-economic context, the sustainable development of cities is very important [1]. Therefore, one of the main challenges presently faced by the building sector is the ageing and early deterioration of structures. This is because structures’ service lives play a significant role in saving resources and reducing not only the carbon footprint, but also building and demolition waste [1]. Indirectly, the rise in structures’ service lives reduces energy use and heat emissions, which are some of main objectives in Horizon 2030 for driving societies to achieve smart and resilient structures for smart cities.

In order to improve the durability of structures, it is necessary to identify and understand their main deterioration precursors. Wide-ranging studies demonstrate how reinforced concrete structures’ (RCSs) service lives are considerably shortened because of corrosion processes [2,3,4]. This is why being able to control the processes that trigger and promote corrosion processes is fundamental. RCS steel corrosion occurs in two stages: the first is the initiation period and the second is the propagation period (according to the model defined by Tutti, K.) [5]. The initiation period entails the diffusion of aggressive agents inside cementitious matrix, which are depassivation precursors. The propagation period implies active rebar corrosion onset. Water plays a fundamental role during both the aforementioned periods: during the initiation period, the water content in the capillary network influences the diffusion capacity of aggressive ions such as chlorides and gases (O_2_, CO_2_, etc.) [6]. It also affects the kinetics of adverse reactions to durability, such as concrete carbonation [7]. During the propagation period, water is directly involved in iron corrosion reactions [8]. It also plays a fundamental role in concrete’s electric resistivity value, which is directly related to the intensity of macrocell phenomena, in which rebar corrosion rates are known to suddenly rise [9,10,11]. Concrete’s electric resistivity is a specific parameter that indicates concrete’s resistance when electric currents pass through its porous network. Its value depends on water availability, the saline content in the porous network of the cementing matrix and the network’s morphology [12,13]. This is an extremely important parameter when defining which zones are susceptible to suffering accelerated corrosion phenomena. It also allows the estimation of the probability of the intense macrocell effects that may occur, which seriously increase the corrosion rate [12,14].

Therefore, it would be interesting to estimate and monitor over time humidity variation and electric resistivity in hardened concrete to identify critical zones in structures before active corrosion phenomena occur in them.

Many types of sensors can detect humidity variation. Currently, the most widespread conventional methods for measuring humidity are amperometric, voltammetric, potentiometric, capacitive, quartz crystal microbalance (QCM), fiber-optic, surface acoustic wave (SAW) or resonance sensing [15]. The most widely used sensors are the capacitive type, whose estimated share of the whole market is close to 75% [15]. However, the variability of these sensors’ sensitivity can be extremely wide depending on the measured humidity levels [15]. In the literature, we find the very interesting Wireless Humidity Sensor System proposed by Zhou S. et al. [16]. This method appears to be effective and easy to place in real structures. However, it is based on Ultrahigh Frequency (UHF) and Radio Frequency Identification (RFID). Recently researchers have proposed synthetic aperture radar imaging to monitor the humidity level of concrete, such as the approaches of Alzeyadi A and Yu T. [17,18]. Both these techniques are more complex than that based on electrochemical technology and operators should have specific knowledge [19].

Studies about using piezoelectric and smart aggregates embedded in concrete structures to detect their deterioration are currently increasing [19]. However, these methods significantly increase the cost of the concrete, and the smart aggregates’ response may be affected by their interference with other substances contained in the concrete pore solution.

To obtain in situ electric resistivity, the most widespread standardized technique today is the so-called four-point method or Wenner’s method (portable Wenner device) [20]. This method is widely used due to its advantages, i.e., it is simple, easy to apply and relatively inexpensive. However, it is a sporadic measuring system that is not designed to be incorporated into a monitoring system. Moreover, its repeatability and reproducibility are low compared to other methods that employ embedded elements; for instance, that presented by Priou J. et al. [12], whose system is efficient, but whose information is limited to resistivity. Most recently, the embedded sensor system presented by Ramon Zamora et al. [21] allows the concrete resistivity and corrosion rate of RCSs to be measured.

It is necessary to bear in mind that most of the aforementioned sensors and systems are not designed to be placed inside monitoring systems formed by a network of smart sensors. It is envisioned that distributed multiparameter sensors have the advantage of reducing the resources used in the system by decreasing the material and economic cost, which can also greatly improve structure control because the number of monitored variables is bigger and, therefore, allows more robust prediction and estimation models to be developed. This agrees with the vision of smart structures for smart cities based on the use of smart sensor networks.

In order to obtain a suitable smart monitoring system of structures, the following system commitment points can be defined:

(1) detecting and verifying if any damage is, or aggressive agents are, present in the structure;
(2) locating any risk zones;
(3) estimating/quantifying harm or the presence of aggressive agents;
(4) making a prognosis of, or predicting, the structure’s service life under these conditions.

In order to develop a network of smart sensors, setting up stainless steel (SS) voltammetric sensors in such a network is very interesting, as demonstrated in former studies [22,23,24,25]. Moreover, SS is an economic and resistant material that allows sensors to be manufactured with bigger effective surface areas and at a lower cost than with other noble metals such as Au and Pt, which have been traditionally used to manufacture voltammetric sensors [26,27].

The present study focused on developing two estimation models by means of a SS sensor system’s response. The estimation models were an estimation model of humidity variation and an estimation model of concrete’s electric resistivity. To develop both, a system in concrete samples submitted to different humidity conditions was calibrated. Next, the system was fitted to four different mathematical models, and the most significantly reproduced reality was selected. Finally, models were validated. During validation, both models explained over 80% of variance.

## 2. Experimental

### 2.1. Sensor System

The employed voltammetric sensors systems were the SS type, whose response has already been studied by the researcher group in previously published studies [22,23].

#### 2.1.1. Electro-Analytical Techniques

The followed electro-analytical technique was impedance spectroscopy (EIS), and the wand was applied using Autolab PGSTAT10 equipment. Data were collected by the Nova 1.11. software.

EIS was applied using the two-electrode configuration, where the sensor acted as the working electrode (WE) and the SS meshing as the pseudo-reference/counter electrode (CE).

This configuration was selected to develop a more sustainable monitoring system because it reduces not only the system’s maintenance tasks (by eliminating the reference electrode), but also the energy used by the system. This is because the energy that must be compensated is that directly required to establish the setting potential between the WE and the CE (ΔV_2electrodes_). However, when working with three electrodes, the required energy is that needed to establish a difference in potential between the WE and the CE that allows the difference in the setting potential between the WE and the reference electrode (ΔV_3electrodes_) to remain. This will give ΔV_3electrodes_ > ΔV_2electrodes_. This means that the required three-electrode electric work will be greater and, therefore, more energy will be used. The studies conducted by the researcher team demonstrated that the two-electrode technique was reliable as long as the employed CE surface area was 40-fold bigger than that of the WE [19].

**Figure 1:**
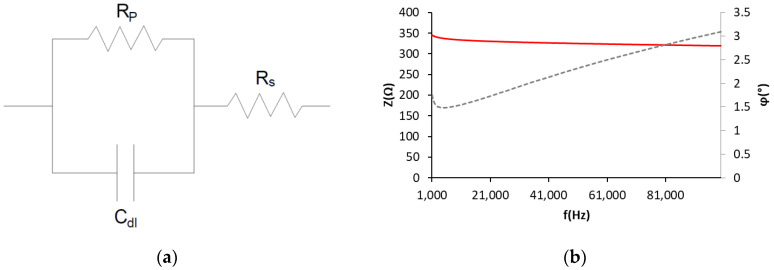
(**a**) Randles electric circuit (R_s_-R_p_/C_dl_). (**b**) Example of the results obtained in the EIS test with the sensors. Z impedance, φ phase of impedance.

The range of sweeping frequencies in EIS went from 100,000 to 1000 Hz. High-frequency sweeping was justified by simplifying the system similarly to the simple equivalent Randles electric circuit (Rs-Rp/C_dl_) (Figure 1a) [24,25]. The applied efficacious stress value was 10 mV. The Rs value was obtained with this test, which was the value of resistance to the ionic circulation at the heart of the electromagnetic field produced between the WE and the pseudo-reference/CE [25].

Figure 1b provides an example of the results obtained with the sensors embedded in concrete by means of EIS. The average phase of the equivalent circuit impedance obtained with sensors was 4.5 ± 3°; therefore, the resistant part of impedance prevails over the reactance part (due to capacitance). In addition to understanding that the capacitor’s impedance is nearly zero at high frequencies (Equation (1)), and is parallel to Rp, all the current flows through the branch of C_dl_. Therefore, under these conditions, the Z equivalent of the circuit equals Rs (Figure 1a).

(1) $$X_{C}=\frac{1}{2\pi \mathrm{fC}}$$

where f is the capacitor’s frequency (Hz) and C is its capacitance.

#### 2.1.2. Electrodes

**Figure 2:**
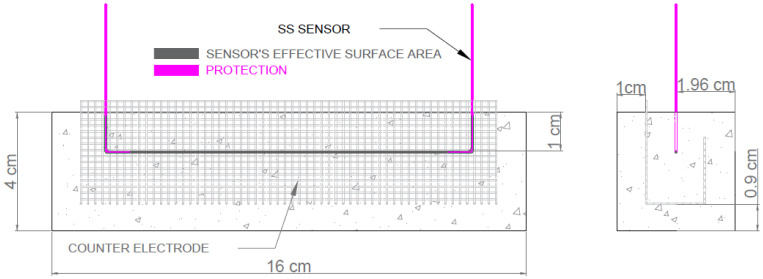
Diagram of the configuration of the SS sensors embedded in a concrete sample.

The employed SS working electrode consisted of a U-shaped SS wire (0.8 mm diameter). The effective working area was limited by covering ends with waterproof polyurethane paint. The average sensor effective surface area was 2.99 ± 0.05 cm^2^ (Figure 2). The sensor’s design effectiveness has been evaluated in former works [23,26].

The pseudo-reference/CE employed to apply electro-analytical techniques was L-shaped SS wire meshing (0.5 mm diameter). Its effective surface area was 132 ± 11 cm^2^.

### 2.2. Methodology

The performed experimental study consisted in developing the following estimation models:

- The estimation model of humidity variation: this was obtained with the correlation of the electric resistance (Rs) data obtained with the weight variation of samples after submitting them to different humidity conditions.
- The estimation model of concrete’s electric resistivity: this was obtained with the correlation of the empirical electric resistivity (ρ) and electric resistance (Rs) data obtained with the sensor system.

To develop both these models, it was necessary to submit them to calibration, fitting and validation processes.

### 2.3. Samples and Materials

In order to develop the estimation models, prismatic concrete samples (4 × 4 × 16 cm^3^) were made with six conventional concrete types without additions, which are specified in Table 1.

To ensure that the study would be statistically reliable, three concrete mixings per concrete type, and four samples per concrete mix, were prepared. The sensor system was embedded in two, but not in the other two. This gave six samples with sensors (CSNS) and six without sensors (SSNS) per concrete, which totaled 72 samples for this study.

For both the CSNS and SSNS samples, four samples per concrete were used to calibrate models, with two samples for concrete validation purposes.

Sensors were placed in the position indicated in Figure 2 to ensure:

- the humidity variations in the environment would quickly affect the zone where sensors were;
- sensors were not affected by any uncontrolled defects on concrete surfaces (hollows, etc.).

### 2.4. Preparing Samples

After concreting, samples were left for 48 days inside a curing chamber under conditions with relative humidity (RH close to 100%). After this time, they were taken out of the chamber and immersed in water to maintain the saturation conditions. Tests were carried out under these conditions on 3 consecutive days.

When tests finished, the samples with sensors (CSNS) and without sensors (SSNS) were left inside an oven at T ≈ 50 °C for 48 h to be dried. After this 48 h period, all the samples were allowed to cool and were equilibrated in a drier with humidity-controlling salt to avoid samples absorbing water vapor while cooling. After 24 h, tests were run under these conditions.

Next, all the samples were placed inside closed containers with saturated salt solution to maintain constant RH conditions with RH values of around 100% [27]. The temperature conditions were those of the laboratory (T = 22 ± 2 °C). Samples were left under these conditions before tests began, and until the variation in their weight between two weighing sessions on 2 consecutive days equaled or went below 2%. This preparation allowed samples’ reference weight to be determined to establish the variation in humidity of the correlations (m_0_). Tests began after samples’ weight stabilized.

Finally, samples were left under laboratory conditions for 72 h (RH ≈ 60%, T = 23 ± 2 °C) and the last lot of tests was performed.

### 2.5. Tests

In each state, three measurements were repeated 3 times per sample to ensure the statistical reliability of the results. Samples were weighed after each test.

#### 2.5.1. Electric Resistivity Measurement

**Figure 3:**
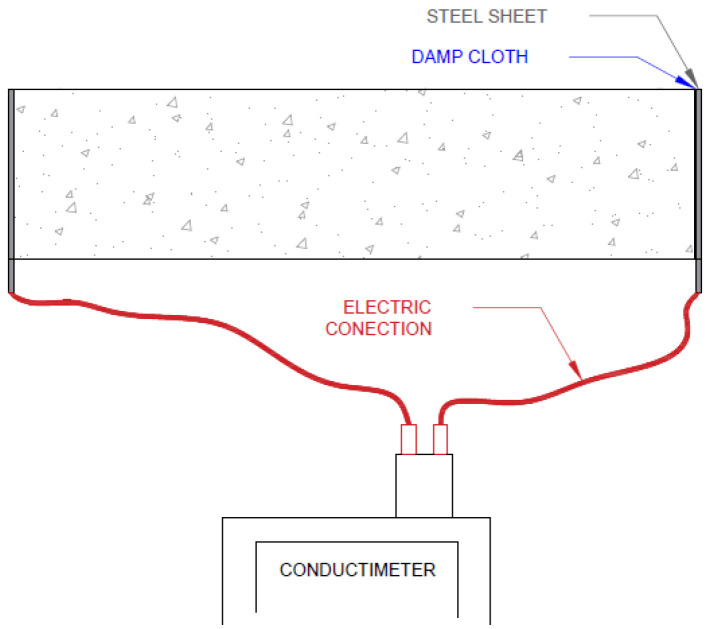
Assembly for measuring with a conductimeter.

The direct method (UNE 83988-1:2008) was applied to obtain samples’ electric resistivity. The measurement was taken by a Knick Portavo 902 conductimeter. This device was connected to two metal plates placed aside samples. The electrical contact between plates and concrete was ensured using a damp cloth. Figure 3 shows the assembly.

Samples’ electrical conductance was obtained using the conductimeter. The inverse of this value was concrete’s electric resistance (R), given by the relation established for a uniform electric field between two rectangular parallel plates (Equation (2)) to obtain concrete’s electric resistivity [28]:

(2) $$\rho =R\;*\;\frac{S}{l}.$$

where ρ is electrical resistivity (Ωm); S is the transversal area of the electric field (m^2^); l is the distances between electrodes (m).

#### 2.5.2. Concrete Characterization Tests

To define the quality of the manufactured concretes, the following concrete characterization tests were conducted:

- Hardened concrete tests. To determine resistance to compression (UNE 12390-3:2009). Resistance to compression was determined at 28 days (fc). Two cylindrical samples were prepared (10 cm diameter, 20 cm high) for each mass at each dose. Total number of samples: 36.
- Determining water absorption, density and accessible porosity for water (UNE 83980:2014). Testing was conducted at 28 days. Two cylindrical samples were prepared (10 cm diameter, 5 cm high) for each mass at each dose. Total number of samples: 36.
- Determining air permeability (UNE 83981:2008). Testing was conducted at 28 days. Two cylindrical samples were prepared (15 cm diameter, 5 cm high) for each mass at each dose. To run the test, sample sides were covered with sealing paint. The air permeability coefficient (k) was obtained. Total number of samples: 36.
- Determining electrical resistivity (ρ): direct (reference) method (UNE 83988-1:2008). Progression over time. Two prismatic samples were prepared (4 × 4 × 16 cm^3^) for each mass at each dose. Total number of samples: 36.
- Determining the water cover depth under pressure (UNE 83-309-90). A test was conducted at 28 days. For this test, two samples were prepared (15 cm diameter and 30 cm high for each mass at each dose. Total number of samples: 36.
- Accelerated chloride diffusion test according to Standard NT-BUILD 492. A test was run at 28 days. Two samples were manufactured (5 cm diameter, 10 cm high) for each mass at each dose. Total number of samples: 36.

## 3. Results and Discussion

### 3.1. Concrete Characterization Tests

Table 2 shows the average values obtained per dose with the characterization tests. The average coefficient of variation is also provided (Avrg Coef.V), where Coef.V is defined as the quotient between the standard deviation and the average value.

After taking into account the results of the characterization tests and the durability indicators set by the French Civil Engineering Association (AFGC) [29], the concretes employed in this work can be classified as follows:

- Very low-durability concretes: w/c = 0.9 and w/c = 0.8. These concretes are not covered by Spanish Standard EHE-08 for their structural use. Their use is justified when the sensor needs to be characterized within a wide range of porosities.
- Low-durability concrete: w/c = 0.6.
- Medium-durability concrete: w/c = 0.5.
- High-durability concrete: w/c = 0.4 and w/c = 0.3.

In line with this, different conventional concrete qualities were included in this study to obtain more reliable results.

### 3.2. Humidity and Electric Resistivity Models

To first establish correlations, the variation in samples’ weight in relation to m_0_ was estimated. This variation was established according to the definition in Equation (3):

(3) $$\frac{\Delta m}{m_{0}}=\frac{m_{i}-m_{0}}{m_{0}}$$

where:

m_0_ is sample weight under these conditions: RH ≈ 100%, T = 22 ± 2 °C. Weight under these conditions was taken as a reference because in a real case, it is believed that the continuous measurement of the humidity state in structures would begin under similar conditions by taking into account the mean year-long temperature in Spain (kg).

m_i_ is the sample weight in a given measurement number (kg).

This variation in the concrete sample’s weight is directly associated with the loss or gain of the water inside it because there are neither salts that can diffuse to a sample nor variations in the temperature that can affect its weight.

#### 3.2.1. Estimation Model of Humidity Variation

**Figure 4:**
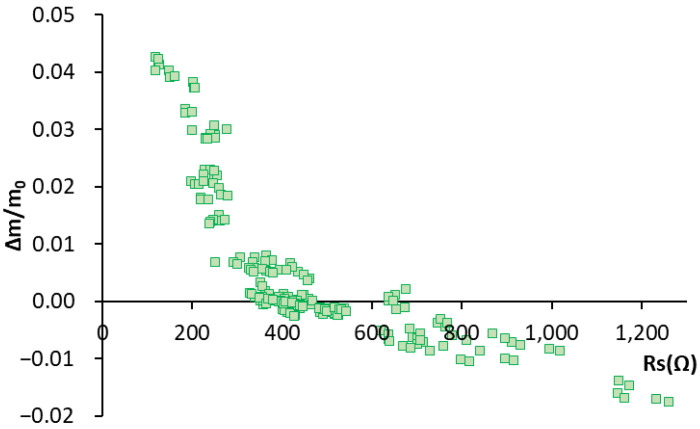
Graphical representation of the humidity variation values vs. the Rs value obtained with the sensor for all the studied concretes.

Figure 4 depicts the humidity variation data for the Rs value obtained by the EIS test by means of SS sensors. We can see an inverse correlation. When humidity variation increases (Δm/m_0_), Rs decreases.

Taking into account the morphology of the correlation and its functions found in the bibliography, three models were used in the calibrating and fitting phases. The first of these was the “modified Stam model” (Equation (4)). This function derives from the equation proposed by Stam, A.J. [30], which was defined for wood by Norberg P. [28]. Its use has been extended to porous building materials. In our case, the equation was modified by adding the sum of a constant, thus improving the fit.

Another fitting function proposed for calibration was the linear correlation between humidity variation and the electric resistance natural logarithm, the “natural logarithm model” (Equation (5)). Different authors resorted to this correlation in previous studies [31,32]. Finally, what we call the “modified common logarithm model” was employed by adding the term “modified” (Equation (6)). We called it a modified model because a constant was added to an already existing model referenced in Machado, J.E. [33].

(4) $$\mathrm{Modified}\;\mathrm{Stam}\;\mathrm{model}\to \frac{\Delta m}{m_{0}}=10^{-q}{\;*\;\mathrm{Rs}}^{k}+p$$

(5) $$\;\mathrm{Natural}\;\mathrm{logarithm}\;\mathrm{model}\to \frac{\Delta m}{m_{0}}=z\;*\;\ln\left(\mathrm{Rs}\right)+s$$

(6) $$\mathrm{Common}\;\mathrm{logarithm}\;\mathrm{model}\;\to \frac{\Delta m}{m_{0}}=\frac{h}{\log\left(R_{s}\right)}+d$$

where q, k, p, z, s, h and d are the unknown constant factors.

The proposed models were solved by the SOLVE tool and making iterations converge until the minimum root-mean-squared deviation (RMSE) value was obtained, as defined according to Equation (7):

(7) $$\mathrm{RMSE}=\sqrt{\frac{\sum {\left({\frac{\Delta m}{m_{0}}}_{\mathrm{calculate}}-{\frac{\Delta m}{m_{0}}}_{\mathrm{real}}\right)}^{2}}{n}}$$

where n is the number of samples.

**Figure 5:**
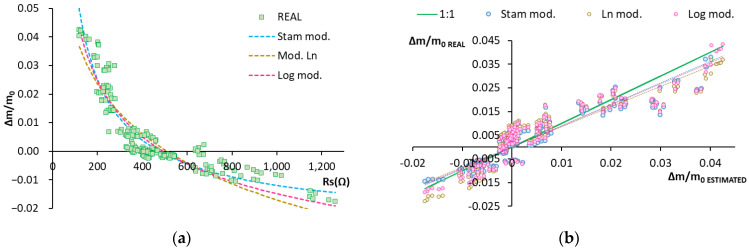
Model of humidity variation according to Rs. (**a**) Graphical representation of the real data vs. those obtained with models. Real data: green squares. Stam model: dashed blue line. Natural logarithm model: dashed yellow line. Common logarithm model: dashed pink line; (**b**) Graphical representation of real data vs. estimated ones. Stam model: blue dots. Natural logarithm model: yellow dots. Common logarithm model: pink dots.

Figure 5a shows the experimental values and the fits of function Δm/m_0_ = f(Rs) for the different models. The Stam model seems to have best represented reality.

Figure 5b depicts the correlation of the estimated humidity variation values versus the real ones. For purposes of interpreting the graph, the slope of the fit lines was used (which indicates the grade of coincidence between the measurement and estimate data), as was the R^2^ value (which explains the percentage of variation in the estimated data vs. the measured data). The different models’ fit was good because the fitted lines came very close to the 1:1 line with little scattering for the dots (slope values close to 0.9 (real vs. estimated line slope) (Table 3)). R^2^ was over 0.8 in all cases (Table 3). This means that the percentage of variance accounted for by the models was at least 80%.

The model with the lowest RMSE value was selected, and the highest slope and R^2^ value was chosen. This corresponded to the Stam model (Equation (8)):

(8) $$\frac{\Delta m}{m_{0}}=10^{-0.403}{\;*\;\mathrm{Rs}}^{-0.727}-0.028$$

##### Model Validation

After selecting the model to which the experimental data best fitted, validation was undertaken using the data of the samples not utilized for the model’s calibration fit. In the sample group for validation, there were two samples per concrete, which were exposed to the same states as the samples used in fitting and calibrating.

**Figure 6:**
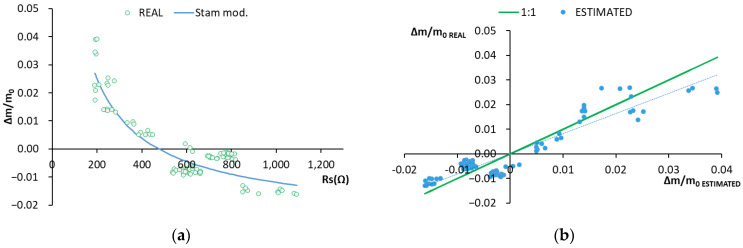
(**a**) Graphical representation of the real validation data and those obtained with the Stam model. Real data: green circles. Stam model: blue line. (**b**) Graphical representation of the real data vs. those estimated. Real data 1:1 line: green line. Stam model: blue dots.

Figure 6a shows the real values employed in the validation (green circles) and the values estimated with the model (blue line). To obtain the estimated Δm/m_0_ data, the Rs values obtained in the validation group of samples were substituted in Equation (7).

Figure 6b depicts the scatter plot of the real vs. estimated values. The fit line of these values has a slope of 0.818, which comes quite close to line having a slope of 1. R^2^ indicates that the model represents 83.5% of the data variance (Figure 6; Table 4).

As work was undertaken with heterogeneous material, the values obtained in the RMSE validation on the real line slope vs. the estimated one and R^2^ were considered acceptable.

#### 3.2.2. Estimation Model of Concrete’s Electric Resistivity

The developed sensor system yielded the Rs value for resistance, which opposed the system’s electrolyte (concrete in this case) when electrical charges passed through it. This parameter was neither specific nor standardized, and depended on the cell constant of the electric field generated between the WE and the pseudo-reference CE. Thus, it could not be compared among different systems.

Given the sensor system’s electrodes configuration, the applied field was not uniform and, thus, the expression defined in Equation (1) could not be used, which corresponded to a uniform electric field between two parallel plates.

Therefore, to obtain a specific value that would allow us to compare different zones and configurations, a decision was made to empirically seek the existing correlation between ρ and Rs.

**Figure 7:**
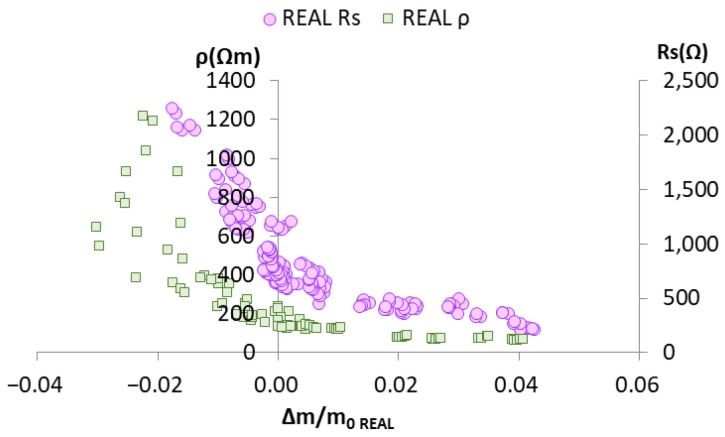
Graphical representation of the Rs values obtained with the sensors vs. real humidity variation in the corresponding samples (lilac circles) and a graph of the measured ρ values in the samples vs. real humidity variation in the corresponding samples (green squares).

To do so, first the graphical representation of the real Δm/m_0_ vs. ρ_REAL_ and the graphic Δm/m_0_ vs. the Rs obtained with the sensor were compared (Figure 7). The morphology of the curves defining the scatter dots was similar. A correlation was established between ρ and Rs by the functions that defined its relation to humidity variation [Δm/m_0_ = f(ρ) α Δm/m_0_ = f(Rs)].

Thus, to obtain the equation with the best fit Δm/m_0_ = f(ρ), the similarity seen in Figure 7 was taken into account and the same fitting models were used as for model Δm/m_0_ = f(Rs).

**Figure 8:**
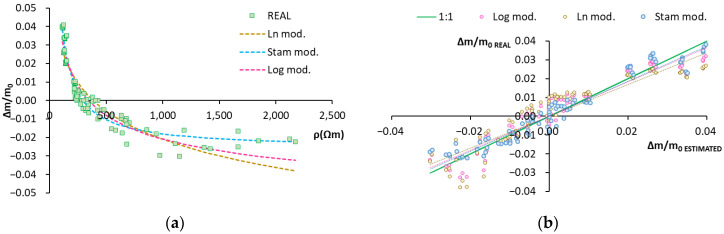
Model of humidity variation according to electric resistivity. (**a**) Graphical representation of the real data and those obtained with the models. Real data: green squares. Stam model: dashed blue line. Natural logarithm model: dashed yellow line. Decimal logarithm model: dashed pink line. (**b**) Graphical representation of the real data vs. the estimated ones. Stam model: blue dots. Natural logarithm model: yellow dots. Decimal logarithm model: pink dots.

Figure 8a includes the values of function Δm/m_0_ = f(ρ), the real values and those obtained with the models. The Stam model appears to better represent reality, as does model Δm/m_0_ = f(Rs).

Figure 8b shows the correlation of the estimated humidity variation values versus the real ones. The different models’ fit is good. The fitted lines come very close to the 1:1 line and the dots are barely scattered. The values of the slopes are around 0.94, except that of the Ln model, which is about 0.84 (Table 5). This indicates a correlation close to 94% between the real and estimated values for all the models, and a slightly lower correlation of 84% for the Ln model. The R^2^ values (Table 5) are around 0.94, except that of the Ln model, which is around 0.83. This indicates that the models account for at least 94% of data variance, except the Ln model, which accounts for 83%.

Bearing in mind the parameters selected to analyze the statistical goodness of fit (RMSE, real line slope vs. the estimated one, and R^2^), the modified Stam model was selected (Equation (9)).

(9) $$\frac{\Delta m}{m_{0}}=10^{-0.745}{\;*\;\rho }^{-0.942}-0.026$$

Finally, to obtain the estimation model of concrete’s electric resistivity using the electric resistance obtained with the sensor’s Rs, Equations (8) and (9) were derived. This equality was simplified by finding ρ according to Rs and solving. This was how Equation (10) was obtained. It defines the correlation between ρ and Rs. This correlation demonstrates that the field generated in the sensor system case was not a uniform field. Therefore, the simplifications related to this condition could not be applied.

(10) $$\rho =2.305{\;R}_{s}{}^{0.772}$$

##### Model Validation

**Figure 9:**
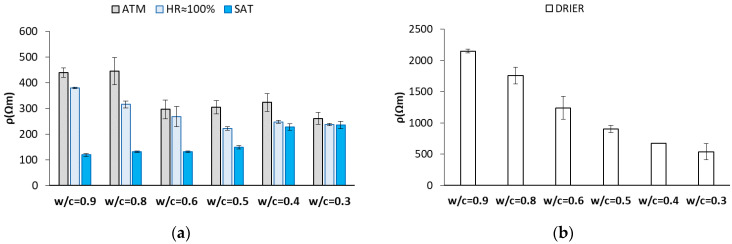
Average electric resistivity values obtained in the validation samples without sensors. (**a**) ATM: laboratory humidity and temperature conditions. HR ≈ 100%: reference condition for weights. SAT: samples immersed under the water condition. (**b**) Drier: condition of the samples dried at 50 °C and left in the drier to cool with salt.

For validation, no samples in which concrete’s electric resistivity was measured by the direct method, and in which electric resistivity was measured with sensors, were available because the presence of the sensor system would have disturbed the measurement taken by the direct method. This is why the average resistivity value obtained in the SSNS samples (not used in the calibration or fitting stages) was employed for the validation per concrete and per state (Figure 9). In the sample group for validation, there were two samples per concrete, which were submitted to the same states used in fitting and calibrating.

**Figure 10:**
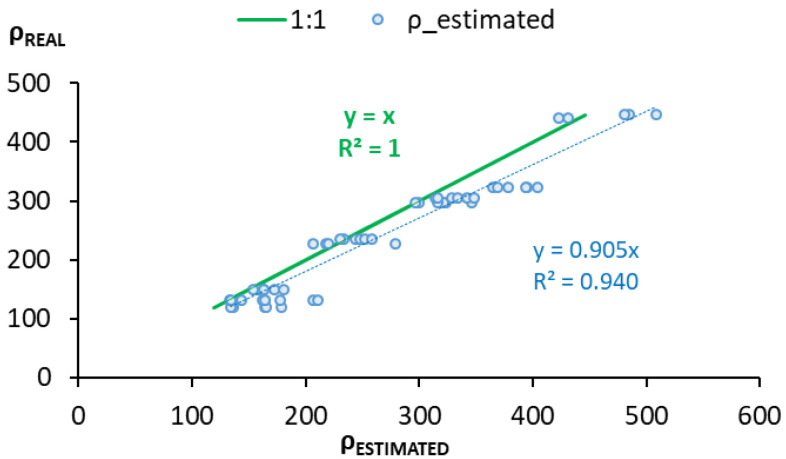
Graphic representation of the real vs. estimated data. Real data 1:1 line: green line. ρ = f(Rs): blue dots.

Figure 10 represents the scatter plot of the real vs. estimated value. The ρ estimated data were obtained by replacing the experimental values of Rs in Equation (10). The fitted line of these values has a slope of 0.905 (Table 6), and this value means 90.5% coincidence between the data. R^2^ indicates that the model represents 94% of the data variance (Table 6).

As work was undertaken with heterogeneous material, the values obtained in the RMSE validation, the real vs. the estimated line slope, and R^2^ could be considered acceptable. The model precisely reproduced reality.

## 4. Conclusions

This paper reviewed research on developing models with an SS sensor system for hardened concrete’s humidity variations and concrete’s electric resistivity. These parameters play a key role in RCS durability. Based on the above review, the following conclusions can be drawn:

- The developed sensor system allows a reliable estimation model of concrete’s humidity variation to be established. It accounts for 88.9% of data variance.
- As Δm/m_0_ = f(ρ) and Δm/m_0_ = f(Rs) functions have the same curve morphology, their empirical data fit the same function typology.
- This study demonstrates how the electric field generated in the sensor system cannot be considered uniform because no uniform linear correlation exists between electric resistivity and electric resistance. This means that uniform field simplifications cannot be used to achieve electric resistivity by means of the sensor system.
- With the correlation between functions Δm/m_0_ = f(ρ) and Δm/m_0_ = f(Rs), the estimation model of electric resistivity by the parameter is obtained with the sensor system’s Rs. This model offers good reproducibility of reality by accounting for 94% of data variance.
- Working with two electrodes implies there are no limitations to the developed models’ reliability and reduces the energy needed for the system.

Based on the above review, the following opportunities for future research into this sensor to monitor the RCS state can be identified:

• The sensor system presented with the developed models can be coupled to a multi-sensor system, developed according to the smart sensor network concept. Depending on data requirements about the state, this sensor network is capable of collecting interesting data at the necessary points to optimize the system’s data resources, and its economic and energy uses.
• It is possible to collect further information from the electric resistivity estimation to characterize the degree of corrosion with these relations:
✓ The correlation between the electric resistivity value and the presence of chlorides. Initial tests can be conducted to check if the model slightly varies when chloride anions are present in the concrete’s porous solution.
✓ The correlation between concrete’s electric resistivity and the corrosion electric current, which are correlated by Ampère’s Law.

## Figures and Tables

**Table 1:** Composition of the used concretes.

Materials	kg/m^3^_concrete_					
	w/c = 0.9	w/c = 0.8	w/c = 0.6	w/c = 0.5	w/c = 0.4	w/c = 0.3
I 42.5 R-SR5 cement	225	250	315	385	490	650
Water	203	200	189	193	196	195
Superplasticizer	1.6	1.8	2.2	2.7	3.4	4.6
Silica sand	1433	1431	1212	1179	1115	1108
Gravel	478	477	653	635	601	475

w/c: water/cement ratio; kg/m^3^: kg of materials per m^3^ of concrete.

**Table 2:** Average values of the standardized test results.

	f_c,28days_ (MPa)	% Abs. Water	% P.A.W	P.W.P (mm)	k (×10^−18^ m^2^)	ρ (Ωm)	D_nssm_ (×10^−12^ m^2^/s)
w/c = 0.9	15.46	8.65%	18.38%	150	996.44	135.87	72.71
w/c = 0.8	18.98	8.38%	18.24%	103	432.15	170.36	50.04
w/c = 0.6	30.74	7.82%	17.00%	18	258.84	178.89	45.22
w/c = 0.5	40.50	7.49%	16.42%	18	50.37	195.69	25.35
w/c = 0.4	60.94	6.58%	14.63%	8	5.68	213.22	9.70
w/c = 0.3	73.52	5.65%	12.80%	0	0.00	281.69	3.73
Avrg Coef V.	4.24%	4.45%	3.86%	6.99%	17.33%	7.87%	4.24%

Resistance to compression at 28 days (f_c,28days_) (UNE 12390-3:2009), % absorbed water (Abs.Water) and % porosity accessible to water (P.A.W) (UNE 83980), penetration depth of water under pressure (P.W.P) (UNE 83-309-90), air permeability coefficient (k) (UNE 83981) and electric resistivity (ρ) (UNE 83988-1:2008), Non-steady-state migration coefficient (D_nssm_) (NT BUILD 492).

**Table 3:** Statistical parameters for evaluating the goodness of the model used in the fit of Δm/m_0_ = f(Rs).

	RMSE	Line Slope Real vs. Estimated	R^2^
Stam mod.	0.0046	0.906	0.889
Ln mod.	0.0057	0.855	0.826
Log mod.	0.0050	0.891	0.870

**Table 4:** Statistical parameters to evaluate the validation of the estimation model of humidity variation in hardened concrete.

	RMSE	Line Slope Real vs. Estimated	R^2^
Stam mod.	0.0053	0.818	0.835

**Table 5:** Statistical parameters to evaluate the model’s goodness of fit of Δm/m_0_ = f(ρ).

	RMSE	Line Slope Real vs. Estimated	R^2^
Stam mod.	0.0045	0.944	0.941
Ln mod.	0.0075	0.842	0.832
Log mod.	0.0059	0.932	0.939

**Table 6:** Statistical parameters to evaluate the validation of the estimation model of concrete’s electric resistivity.

	RMSE	Line Slope Real vs. Estimated	R^2^
Stam mod.	36.444	0.905	0.940

## Data Availability

Not report any data.

## References

[1] Naik T.R. (2008). Sustainability of Concrete Construction. Pract Period Struct Des. Constr..

[2] Biswas R.K., Iwanami M., Chijiwa N., Uno K. (2020). Effect of non-uniform rebar corrosion on structural performance of RC structures: A numerical and experimental investigation. Constr. Build. Mater..

[3] Fernandez I., Berrocal C.G. (2019). Mechanical Properties of 30 Year-Old Naturally Corroded Steel Reinforcing Bars. Int. J. Concr. Struct. Mater..

[4] Almusallam A.A. (2001). Effect of degree of corrosion on the properties of reinforcing steel bars. Constr. Build. Mater..

[5] Tutti K. (1982). Corrosion of Steel in Concrete.

[6] Linares Alemparte P. (2015). Caracterización del Hormigón en Relación a la Difusión de Gases y su Correlación con el Radón. Ph.D. Thesis.

[7] Lee H.M., Lee H.S., Min S.H., Lim S., Singh J.K. (2018). Carbonation-induced corrosion initiation probability of rebars in concretewith/without finishing materials. Sustainability.

[8] Singh D.V., Sachan A.K., Rawat A. (2016). Developments in corrosion detection techniques for reinforced concrete structures. Indian J. Sci. Technol..

[9] Laurens S., Hénocq P., Rouleau N., Deby F., Samson E., Marchand J., Bissonnette B. (2016). Steady-state polarization response of chloride-induced macrocell corrosion systems in steel reinforced concrete—Numerical and experimental investigations. Cem. Concr. Res..

[10] Hansson C.M., Poursaee A., Laurent A. (2006). Macrocell and microcell corrosion of steel in ordinary Portland cement and high performance concretes. Cem. Concr. Res..

[11] Elsener B. (2002). Macrocell corrosion of steel in concrete—Implications for corrosion monitoring. Cem. Concr. Compos..

[12] Priou J., Lecieux Y., Chevreuil M., Gaillard V., Lupi C., Leduc D., Rozière E., Guyard R., Schoefs F. (2019). In situ DC electrical resistivity mapping performed in a reinforced concrete wharf using embedded sensors. Constr. Build. Mater..

[13] Azarsa P., Gupta R. (2017). Resistivity of Concrete for ElectricalDurability Evaluation: A Review. Adv. Mater. Sci. Eng..

[14] Feliu S., Andrade C., González J.A., Alonso C. (1996). A new method for in-situ measurement of electrical resistivity of reinforced concrete. Mater. Struct. Constr..

[15] Buasiri T., Habermehl-Cwirzen K., Krzeminski L., Cwirzen A. (2021). Novel humidity sensors based on nanomodified Portland cement. Sci. Rep..

[16] Zhou S., Deng F., Yu L., Li B., Wu X., Yin B. (2016). A novel passive wireless sensor for concrete humidity monitoring. Sensors.

[17] Alzeyadi A., Yu T. (2018). Moisture determination of concrete panel using SAR imaging and the K-R-I transform. Constr. Build. Mater..

[18] Alzeyadi A., Yu T. (2020). Subsurface characterization of moisture content and water-to-cement ratio of concrete specimens using remote synthetic aperture radar imaging Subsurface characterization of moisture content and water-to-cement ratio of concrete specimens using remote synthetic aperture radar imaging. J. Appl. Remote Sens..

[19] Zhang H., Li J., Kang F. (2022). Real-time monitoring of humidity inside concrete structures utilizing embedded smart aggregates. Constr. Build. Mater..

[20] Ghods P., Alizadeh A.R., Salehi M. (2015). Electrical Resistivity of Concrete. Concr. Int..

[21] Ramón J.E., Martínez I., Gandía-romero J.M., Soto J. (2021). An embedded-sensor approach for concrete resistivity measurement in on-site corrosion monitoring: Cell constants determination. Sensors.

[22] Martínez-Ibernón A., Roig-Flores M., Lliso-Ferrando J., Mezquida-Alcaraz E., Valcuende M., Serna P. (2020). Influence of cracking on oxygen transport in UHPFRC using stainless steel sensors. Appl. Sci..

[23] Martínez-Ibernón A., Lliso-Ferrando J., Gandía-Romero J.M., Soto J. (2021). Stainless Steel Voltammetric Sensor to Monitor Variations in Oxygen and Humidity Availability in Reinforcement Concrete Structures. Sensors.

[24] Ramón Zamora J.E. (2018). Sistema de Sensores Embebidos para Monitorizar la Corrosión en Estructuras de Hormigón Armado. Fundamentos, Metodología y Aplicaciones. Ph.D. Thesis.

[25] Martínez-Ibernón A., Ramón J.E., Gandía-Romero J.M., Gasch I., Valcuende M., Alcañiz M., Soto J. (2019). Characterization of electrochemical systems using potential step voltammetry. Part II: Modeling of reversible systems. Electrochim. Acta.

[26] Martinez Ibernon A., Gasch I., Romero J.M.G., Soto J. (2022). Hardened Concrete State Determination System Based on a Stainless Steel Voltammetric Sensor and PCA Analysis. IEEE Sens. J..

[27] ASTM COMPASS, ® (2018). Standard Practice for Maintaining Constant Relative Humidity by Means of Aqueous Solutions. ASTM Des. E.

[28] Norberg P. (1999). Electrical measurement of moisture content in porous building materials. Durab. Build. Mater. Compon..

[29] Baroghel-Bouny V., Jacob J. (2004). Groupe de Travail CONCEPTION des Bétons Pour une Durée de vie Donnée des Ouvrages—Indicateurs de Durabilité (France). Conception des Bétons Pour une Durée de vie Donnée des Ouvrages: Maîtrise de la Durabilité vis-à-vis de la Corrosion des Armatures et de L’alcali-Réaction.

[30] Stamm A.J. (1927). The Electrical Resistance of Wood as a Measure of Its Moisture Content. Ind. Eng. Chem..

[31] de Medeiros-Junior R.A., da Munhoz G.S., de Medeiros M.H.F. (2019). Correlations between water absorption, electrical resistivity and compressive strength of concrete with different contents of pozzolan. Rev. Alconpat..

[32] Kazmi D., Qasim S., Siddiqui F.I., Azhar S.B. (2016). Exploring the Relationship between Moisture Content and Electrical Resistivity for Sandy and Silty Soils. Int. J. Eng. Sci. Invent..

[33] Machado J.E.O. Características Físico Mecánicas y Análisis de Calidad de granos. Univ. Nacional de Colombia. https://books.google.com.co/books?id=2DWmqb6xP3wC.

